# Lexicalisation and de-lexicalisation processes in sign languages: Comparing depicting constructions and viewpoint gestures

**DOI:** 10.1016/j.langcom.2012.09.004

**Published:** 2012-10

**Authors:** Kearsy Cormier, David Quinto-Pozos, Zed Sevcikova, Adam Schembri

**Affiliations:** a Deafness, Cognition & Language Research Centre, University College London, UK; b University of Texas at Austin, USA; c La Trobe University, Melbourne, Australia

**Keywords:** Classifier, Sign language, Gesture, Point of view, Iconicity

## Abstract

In this paper, we compare so-called “classifier” constructions in signed languages (which we refer to as “depicting constructions”) with comparable iconic gestures produced by non-signers. We show clear correspondences between entity constructions and observer viewpoint gestures on the one hand, and handling constructions and character viewpoint gestures on the other. Such correspondences help account for both lexicalisation and de-lexicalisation processes in signed languages and how these processes are influenced by viewpoint. Understanding these processes is crucial when coding and annotating natural sign language data.

## Highlights

► We compare depicting constructions (DCs) in sign languages (SLs) and gesture. ► We find similarities between entity constructions and observer viewpoint gestures. ► We find similarities between handling constructions and character viewpoint gestures. ► In SLs, lexicalisation and de-lexicalisation in DCs are influenced by viewpoint.

## 1 Introduction

A number of researchers have suggested that sign language grammars include morphosyntactic constructions that can be compared to spoken language classifier constructions (e.g., Emmorey, 2003). The earliest suggestions that signed languages contain forms that are akin to spoken language classifiers focused on the handshapes of particular signs and how those handshapes appeared to categorise referents in particular ways similar to the nominal categorisation seen in spoken language classifier or classificatory verb systems (e.g., Frishberg, 1975; Kegl and Wilbur, 1976; Supalla, 1978). Since the 1990s, however, some of the assumptions that led to the use of the term ‘classifier’ and ‘classifier construction’ for signed languages have been challenged (Cogill-Koez, 2000; Edmondson, 1990; Engberg-Pedersen, 1993; Liddell, 2003b; Schembri, 2001; Slobin et al., 2003), although others have defended this analysis (Supalla, 2003; Zwitserlood, 2003, 2012). Terminology and taxonomy are major factors in the debate, further complicated by the role of iconic and gestural properties within these constructions in signed languages, which some argue play an important role (e.g., Cogill-Koez, 2000; DeMatteo, 1977). For ease of exposition, to refer to the entire range of this set of forms in signed languages, we use the term depicting constructions (DC), following terminology introduced by Liddell (2003a). We use the term depicting handshape units or depicting handshapes to refer to the hand configurations used within depicting constructions rather than ‘classifier’.

In this paper, we explore the issues related to the appropriate characterisation of depicting constructions in a detailed examination of these signs and comparable constructions used by non-signers in gesture. Although some detailed comparisons have been made between depicting constructions and spoken language classifiers (e.g., Schembri, 2003), a similarly detailed comparison between these constructions and specific types of gesture used by non-signers would help further our understanding of the linguistic and gestural processes at work within these constructions in signed languages. Such comparison highlights the key factors that play a part in the different lexicalisation processes involved with entity and handling constructions. We intend to show that understanding these processes is crucial when working with natural sign language data; otherwise one runs the risk of attributing lexical status to constructions which are only partially (or not at all) lexicalised or vice versa. This can have substantial consequences when making claims not only about the linguistic versus gestural nature of depicting constructions, but also about the nature of the sign language lexicon more generally.

## 2 Depicting constructions in signed languages

**Fig. 1:**
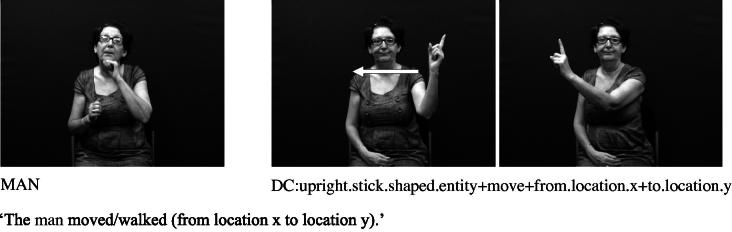
Depicting (entity) construction (BSL).

**Fig. 2:**
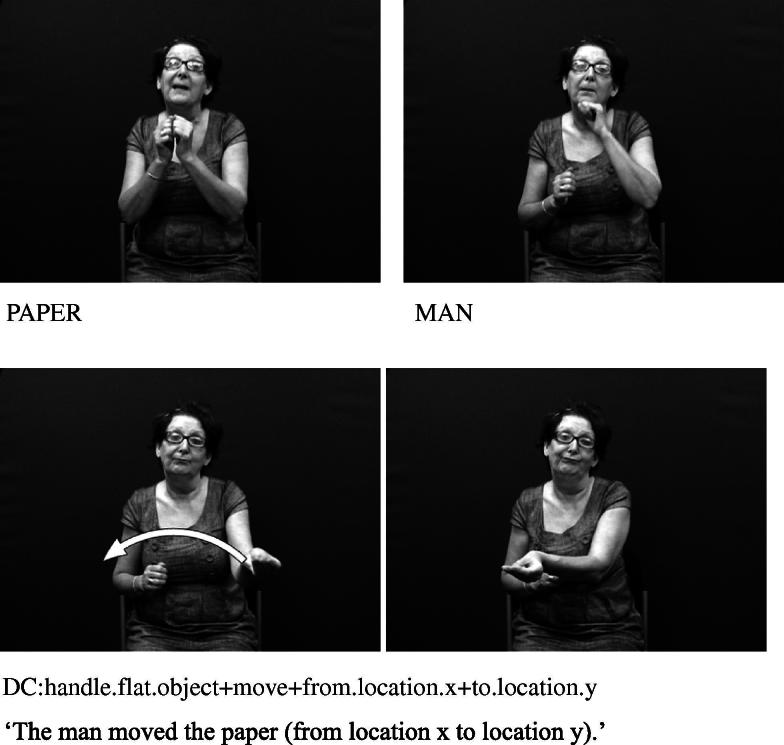
Depicting (handling) construction (BSL).

The core component of the DC has usually been described as the handshape which identifies the class of the referent. Examples from British Sign Language (BSL) in Figs. 1 and 2 show constructions depicting an entity and handling, respectively.^1^ These constructions and the handshape units within them are described in more detail below.

There have been considerable inconsistencies in the terminology used to describe these constructions in the sign language linguistics literature (Schembri, 2003). Frishberg (1975) was among the first to introduce the term ‘classifier’ into the description of signed languages, and Supalla (1978, 1986) was the first to compare classificatory verbs in Athabaskan languages, such as Navajo, to constructions in ASL which involve the motion, location, handling and visual-geometric description of nominal referents. These early claims about the existence of classifier morphemes in American Sign Language (ASL) (e.g., McDonald, 1982; Supalla, 1978) were based on an account of the Navajo classificatory verb system proposed by Allan (1977) which mistakenly identified classifier morphemes in these Navajo forms. (As first pointed out by Engberg-Pedersen (1993), there is no evidence that such morphemes actually exist synchronically in the language.) Since then, claims about similarities between DCs in signed languages and classifiers in spoken language have not gone unchallenged, but they have nevertheless still been widely accepted in the sign linguistics literature, particularly similarities between DCs and verbal classifier systems. Example (1) shows examples of verbal classifiers in the Papuan language Imonda. In these examples, isolatable classifier morphemes (*lëg*- and *u*-, referring to flat objects and small animals, respectively) are attached to the verb stem *aihu*. Verbal classifiers are not as common as other classifier types (e.g. noun or numeral classifiers) but they do occur in some indigenous languages of North and South America and also some Papuan and Australian languages (Aikhenvald, 2003; Grinevald, 2000, 2003). DCs in signed languages (particularly depicting handshapes used within these constructions) do appear to share some characteristics with classifier morphemes in these verbal classifiers (Sandler and Lillo-Martin, 2006; Zwitserlood, 2003), though the extent of similarity is the subject of some debate (see e.g., Schembri, 2003).

(1) Imonda (Papuan; Seiler, 1985, pp. 120-121)

**Table:** 

a.	maluõ	ka-m	lëg-ai-h-u
	clothes	1sg-obj	clf:flat.object-give-ben-imp
	‘Give me a piece of clothing!’		

b.	tõbtõ	ka-m	u-ai-h-u
	fish	1sg-obj	clf:small.animal-give-ben-imp
	‘Give me the fish!’		

**Fig. 3:**
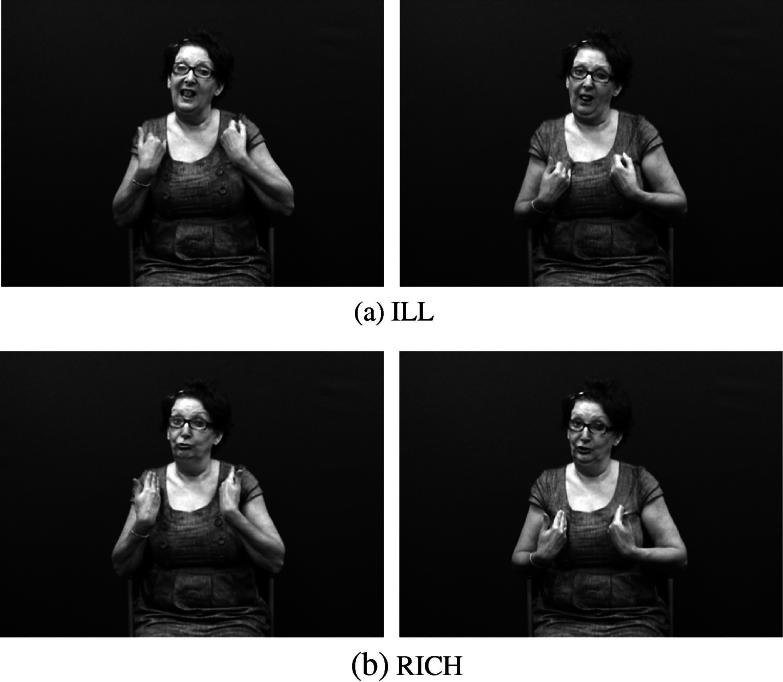
Phonological contrast in BSL (handshape).

**Fig. 4:**
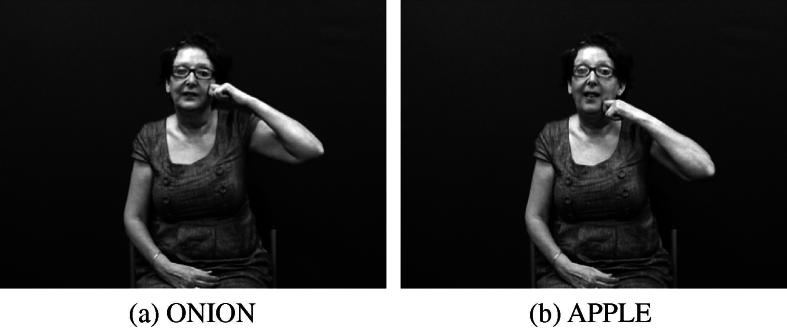
Phonological contrast in ASL (location).

Before examining the various typologies of DCs proposed by sign language researchers, we first briefly introduce the phonological structure of signed languages. Stokoe (1960) first described the sublexical structure of ASL and developed a transcription system based on the contrastive units he identified. These units, now known as the handshape, location and movement parameters of sign production, are contrastive in lexical signs. Thus, the /pinky/ handshape in the BSL sign ILL contrasts with the /flat/ handshape in the BSL sign RICH (both with the same location, i.e. the signer’s chest, and the same downward movement), as shown in Fig. 3. (See also Appendix A for more detailed photos of the /pinky/ and /flat/ handshapes.) Likewise, the location of the ASL sign onion at the temple contrasts with the ASL sign apple at the chin (both with the same handshape, i.e. /intl-T/, and the same movement, i.e. forearm twisting), as in Fig. 4. Handshape, location and movement are the major phonological parameters which have been identified in all signed languages to date, but there are also other minor parameters which may be phonologically contrastive in some signed languages, including palm and finger orientation, hand arrangement (i.e. specifications for one- versus two-handed articulations), and non-manual features. The key parameter for depicting constructions is handshape.

Between two and seven depicting handshape categories have been described for signed languages (see Schembri, 2003 for a summary of the common systems suggested to date). The most basic system is a two-way distinction between forms representing objects and forms representing how objects are handled (Shepard-Kegl, 1985; Zwitserlood, 2003). A tripartite classification (e.g., Brennan, 1992; Engberg-Pedersen, 1993; McDonald, 1982; Schick, 1987; Supalla, 1982) is also common^2^:

I. handshape units that represent a part or the whole of **an object** and have one or both of the following characteristics (common labels include [whole] entity classifiers, limb classifiers, class classifiers, semantic classifiers, static size and shape specifiers [SASSes]):
  a. the object is part of a category whose members are related semantically;
  b. the object possesses certain qualities of size and shape that are matched by aspects of the handshape that is used;
II. handshape units that represent the **handling or manipulation of or manual contact with an object,** i.e. how the hand is configured when handling a particular referent or a part of it (common labels include handle/handling classifiers, touch classifiers)
III. handshape units that contribute to some aspect of a visual-geometric **description of an object** (common labels include tracing classifiers, tracing size and shape [SASS] classifiers, extent classifiers)

The classification outlined above thus separates depicting handshape units in signed languages into three main categories: handshapes which represent objects (either partially or wholly), handshapes which represent handling of objects, and handshapes which describe visual-geometric characteristics of objects. Note that in (III), the handshape unit is part of a description but does not itself represent a referent. In such forms, the handshape may provide some information about the width and depth of the referent, but it is primarily the movement of the hand(s) – not the handshape – that provides information about the size and shape of the object via outlining or tracing the shape (Zwitserlood, 1996). For this reason, following Zwitserlood (2003), our focus will be on the handshapes outlined in (I) and (II). We will not treat the types of handshape forms represented in (III) any further.

Handshape units described in (I), which we refer to collectively as entity handshape units hereafter, are typically understood to represent a whole (or part of a) referent (Engberg-Pedersen, 1993; Liddell and Johnson, 1987). Some researchers also include in this category handshape units known as (static) size and shape specifiers (SASSes) (e.g., Brennan, 1992; Liddell and Johnson, 1987; Supalla, 1986; Zwitserlood, 2003) and instrument classifiers. With static SASSes, the handshape is chosen based on salient characteristics of the referent that the signer wishes to communicate, such as its relative depth and width (e.g., Schembri, 2003). For example, the /flat/ handshape is used in various signed languages to represent objects such as books, vehicles or a surface that can be walked on, as in Figs. 5a and 5b below. The /index/ handshape may be used to represent an upright person or entity that has a stick-like shape, as shown above in Fig. 1 and below in Fig. 5c. Instrument classifiers are used to depict instruments such as tools, utensils or other implements in terms of the physical features of size and shape. For example, the /V/ handshape in BSL can be used to represent instruments such as scissors (Brennan, 1990, 1992), as shown in Fig. 5d.

The handshapes described in (II) – i.e. handling handshape units – represent the way in which a part or all of an object is handled or touched. For example, the /flat-O/ handshape may be used to represent handling of lightweight, flat, thin objects such as a sheet of paper, as in Fig. 2 above (repeated below as Fig. 6a). This category includes handshapes which represent the way objects are touched, such as the /5/ handshape (with finger wiggling) for playing the piano or /index/ for using a calculator, as in Fig. 6b, or the /thumb/ handshape for using a drawing pin. Others such as Brennan (1992) distinguish ‘touch classifier’ as a separate category from ‘handling classifier’.

In addition to issues with terminology and taxonomy, it is also important to note that some sign language researchers reject the notion that depicting handshape units are best considered classifiers in signed languages. This is reflected in the wide variety of terms used to refer to DCs.^3^ Slobin et al. (2003) view DCs as verbs with a handshape which specifies a referent with a particular property; they use the term polycomponential signs for DCs and property marker for the handshape unit. Liddell (2003a) considers at least some types of DCs to be lexical verbs specified for handshape and movement which combine with analogue, gestural elements of location; he uses the term depicting verbs for DCs. Johnston and Schembri (2007) adopt Liddell’s analysis but use a slightly different term – depicting signs – to include those constructions which do not always have a clearly verbal function. Here we use depicting constructions rather than depicting signs to reflect the possibility of more gestural, less lexicalised productions (which may be used by signers or non-signers) versus more lexicalised productions used by signers, as we explain further in our analysis in Section 5 below. Before looking at lexicalisation processes with DCs, we first consider DCs within a model of the sign language lexicon.

## 3 Depicting constructions and the sign language lexicon

**Fig. 7:**
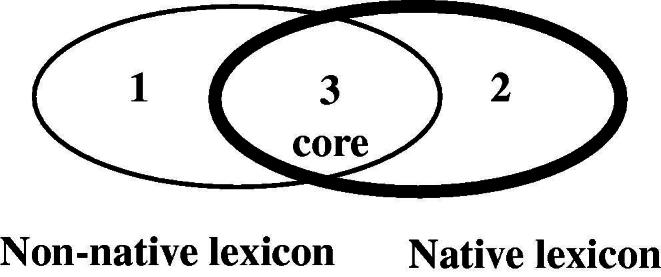
Model of the ASL lexicon (adapted from Brentari and Padden, 2001).

Brentari and Padden (2001) have suggested that the ASL lexicon may be divided into a subcomponent that contains all the native sign vocabulary (called the native lexicon), and a non-native component (the non-native lexicon) that is borrowed from English by means of fingerspelling, as shown in Fig. 7.^4^

Within this model, native signs are signs that have developed within signed languages and conform to a set of constraints, such as the constraint that there may be no more than two types of handshape per sign, first proposed by Battison (1978) for ASL. Non-native forms are lexical items in ASL that include fingerspelled representations of words from the surrounding spoken language – in this case, English. Similar models have been applied/extended to other sign languages including BSL and Australian Sign Language (Auslan) (e.g., Cormier et al., 2008; Johnston and Schembri, 1999).

The native subcomponent of the lexicon may be subdivided into core and non-core components. Fig. 7 shows that the lexicon has three main components, represented by parts 1, 2 and 3. Part 1 represents fingerspelling and signs derived from fingerspelling. Part 2 for Brentari and Padden includes DCs. The central component (3) is the core native vocabulary. Signs may also move between the non-native (1) and the native core (3) lexicon, and between the non-core (2) and the core (3) native lexicon, which is why both parts 1 and 2 overlap with part 3.

Brentari and Padden (2001) refer to the permanent lexicon as the core native lexicon. It is also widely known as the frozen or established lexicon (Brennan, 1992; McDonald, 1985) and corresponds to part 3 of the diagram in Fig. 7. The core native lexicon includes all the permanent items of the sign vocabulary, signs that are highly stable and standardised in form and meaning, and which are used frequently in the language. These signs are known as lexical signs. We can think of lexical signs ‘…as ready-made, off the shelf lexical items. They are already in existence: the signer simply has to pluck them from her/his mental lexicon and place them in the appropriate lexical contexts’ (Brennan, 1992, pp. 45–46).

The distinction between the core, established or frozen lexicon (Part 3 of Fig. 7) and the productive or non-core native lexicon in signed languages (Part 2 of Fig. 7) has been explored in the work of several sign linguists, including Brentari (2001), McDonald (1982), Padden (1998), Johnson and Liddell (1984), Johnston and Schembri (1999), Supalla (1982), and Brennan (1990, 1992, 1994, 2001), although the ways in which this distinction has been described differ considerably. The difference between these two aspects of sign language vocabulary has traditionally been understood in the following way: the core native lexicon (Part 3) consists of those lexical forms which are highly stable and standardised in the language, while the non-core native lexicon (Part 2) is made up of DCs which are highly variable and weakly lexicalised.

The non-core portion of the native lexicon is generally assumed to include both entity constructions and handling constructions. As noted above, non-core native signs may move into the core lexicon over time. For example, as noted by Brentari and Padden (2001), when describing two spacecraft interacting in space (for example, when referring to a science fiction film), an ASL signer might produce a nonce entity construction meaning ‘two aircraft dock (in outer space)’, as demonstrated in Fig. 8a. This construction would derive from a modification of the existing lexicalised verb FLY.BY.PLANE and lexicalised noun sign AIRPLANE in ASL, replicated here in Figs. 8b and 8c. Likewise Aronoff et al. (2003: 69) claim that the ASL sign FALL (as in Fig. 9a for both ASL and BSL) apparently originated as an entity construction in which the hand represents the legs of a two-legged entity (as in Fig. 9b). Over time, they claim, this sign has become more general in its semantic interpretation so that it is no longer restricted to representing only humans but may be used to depict any object falling. Thus, this lexicalised verb may take now apples, boxes or rocks as possible subject arguments in ASL. Thus, as Supalla (1986) also notes, the handshape component of the lexicalised sign FALL no longer has a link to a specific class of referents, despite it iconically representing two-legged entities. The same semantic change has occurred in BSL.

Zeshan (2003: 134) claims that the basic lexicalisation process is essentially the same in handling constructions as well in Indo-Pakistani Sign Language (IPSL). The IPSL sign NEWSPAPER is based on a ‘handling’ construction with a literal meaning that suggests the unfolding of a large flexible object, using a handshape as in Fig. 10b. Zeshan argues that the lexicalisation process has narrowed down the meaning to refer to a newspaper as a lexical item in particular. The BSL sign NEWSPAPER, shown in Fig. 10a, uses the same handling handshapes as described for IPSL.

Although it is clear that entity and handling handshapes do occur in both the non-core lexicon (i.e. DCs) and in the core lexicon (i.e. fixed lexical signs), there is actually very little evidence that this is the result of a historical lexicalisation process in all cases, i.e. lexicalisation in the sense of morphological and syntactic elements moving diachronically into the lexicon (Cabrera, 1998; Hopper and Traugott, 2003). Instead, depicting handshapes (as used in DCs) and lexical signs with depicting handshapes may have co-evolved in lexicogenesis (Zwitserlood, 2003). Thus, the lexical signs FALL and NEWSPAPER shown in Figs. 9a and 10a above may have developed historically at the same time as the corresponding entity and handling handshapes shown in Figs. 9b and 10b. Although there are examples of depicting handshapes that lexicalise within a relatively short time frame in a given signed language so we can see the process occurring (e.g., the BSL sign MOBILE-PHONE developed quite quickly from a handling construction representing the holding of a small, rectangular object near the ear), we do not have enough historical evidence to suggest a particular lexicalisation path for all lexical signs that include a depicting handshape.

Indeed, some researchers have suggested that core lexical items with handling or entity handshapes can also be analysed as depicting constructions within discourse, subject to productive morphological processes which can convey particular aspects of what is being depicted. For example, Aronoff et al. (2003: 74) claim that the Israeli Sign Language (ISL) sign WRITE is a core sign of ISL because it may be modified to indicate temporal aspect inflections, such as continuative aspect, like other lexicalised verbs in the language. However, they note that this sign can be reinterpreted partially as a depicting construction that highlights the signer focusing on the dominant hand representing the hand holding the writing instrument (i.e. a handling construction) and the non-dominant hand representing the object written on (i.e. an entity construction). In such cases, it might be produced as part of a nonce construction with large, sweeping motions to depict someone writing in large letters on a placard, for example. Signers are able to alternate between the articulation of certain core lexical items and decomposed forms which function as related depicting constructions. Indeed, Johnston and Schembri (1999) note that alternations between lexicalised and decomposed (‘de-lexicalised’) forms exist throughout the sign language lexicon. Such an alternation between a core lexical form and a corresponding decomposed form suggests that the lexicalisation pathway with depicting handshapes is not necessarily unidirectional. Rather, this process is similar to grammaticalisation (and de-grammaticalisation) processes where fully grammaticalised elements and corresponding lexical elements co-exist synchronically – both for spoken languages (e.g. ‘gonna’ and ‘going to’ in English; Hopper and Traugott, 2003) and for signed languages (e.g., the grammatical morpheme used to mark future in ASL which has developed from the lexical verb GO; Janzen and Shaffer, 2002).

One question we pose here is whether it is appropriate to have a single unified non-core lexicon (Part 2 in Fig. 7 above) which consists of both entity and handling constructions (and possibly other sign types, such as pointing signs), or whether entity and handling constructions are best considered separate parts of the non-core lexicon.^5^ In handling constructions, the hand represents the hand of the referent while in whole entity constructions the hand represents the entire referent. The scale of perspective is also different; entity constructions represent referents on a small-scale while handling constructions represent referents on a large, real-world scale (Emmorey, 2002; Johnston, 1991; Schick, 1987). With relation to the scale of representation, different types of iconicity are available in entity and handling constructions: while production of handling constructions relies on basic motor and sensory-motor associations which are not mediated by cognitive processes, entity constructions rely on visual-perceptual associations which *are* mediated by cognitive processes (Perniss et al., 2010). In terms of scale and articulator/meaning correspondence, fully lexical versions of entity and handling constructions have (or at least may have) the same iconic properties as respective non-lexicalised versions. Thus, for example, with both the biped entity handshape /V/ and in the lexical sign FALL as in Figs. 9a and 9b, the handshape represents legs of a biped (although as noted above the sign FALL has been semantically extended in some signed languages to include referents which do not have legs – e.g. this sign could be used in BSL to refer to falling leaves). With both the handling handshape /intl-T/ and the IPSL lexical sign NEWSPAPER as in Figs. 10a and 10b, the signer’s hand represents manipulation of an object that has the same dimensions as the edges of a newspaper. Are there ways of representing such iconic correspondences (e.g. the correspondence between /V/ and FALL and the correspondence between /intl-T/ and NEWSPAPER) in the model of the sign language lexicon? We suggest that there are, particularly when considering likely gestural origins of entity and handling constructions.

## 4 Gestural characteristics of depicting constructions in signed languages

One of the main goals of the earliest work on DCs in signed languages was to show that these constructions are linguistic rather than gestural (e.g., Schick, 1987; Supalla, 1978, 1982, 1986). Emphasis was on showing that DCs are conventionalised, combinatorial, and composed of discrete segments similar to proposed defining properties of language, and not as idiosyncratic, unanalysable wholes as pantomime and iconic gesture have been claimed to be (McNeill, 1992). This, in addition to the misunderstanding of Allan’s (1977) analysis of spoken language classifiers as described in Section 1, is one of the reasons why the term ‘classifier’ was adopted from spoken languages to describe the handshape unit in DCs in the first place. Indeed, although researchers disagree about the degree to which DCs are similar or different to classifiers in spoken languages (e.g., Schembri, 2003; Zwitserlood, 2003), a widely held view amongst some sign language researchers is that DCs are quite different from gestures used by non-signers (e.g., Supalla, 2003), even though few studies have explicitly compared them.

Before comparing DCs and gesture, it is important to clarify what we mean by ‘gesture’. There are many different types of gestures used by non-signers (Kendon, 2004; McNeill, 1992). Emblems are gestures which are highly conventionalised, have standards of well-formedness, and have meanings which may vary across cultures – e.g., the thumbs-up gesture for giving approval used in the English-speaking world may also represent the number one in some European nations. Pantomimic or mimetic gesture depicts objects or actions. Emblems and mimetic gesture do not rely on speech and, like emblems, may occur without it, unlike co-speech gesture. McNeill (1992) divides co-speech gestures into several main types: iconics, metaphorics, beats, and deictics. Iconic gestures are those in which the form of the gesture represents a concrete meaning. With metaphoric gestures, the form of the gesture represents an abstract concept. Beat gestures are contentless gestures which usually follow the rhythm of speech, and deictic gestures are those which have a pointing form or function. Of these different gesture types, DCs in signed languages share the most properties with iconic gestures, although metaphoric uses of DCs are also possible (Brennan, 1990; Taub and Galvan, 2001).

McNeill (1992) further breaks down iconic gestures into observer viewpoint gestures and character viewpoint gestures. Character viewpoint gestures are those where the hands and/or other articulators represent the same articulators of the referent. Character viewpoint gestures produced by the hands are clearly analogous to handling constructions in signed languages – i.e. where the signer’s/gesturer’s hands represent the hands of the referent. Observer viewpoint gestures are those in which the hand(s) represent an entire referent; these are analogous to entity constructions in signed languages. Although McNeill considers both observer viewpoint and character viewpoint gestures to be subtypes of iconic co-speech gestures, each of these viewpoints can be used within mimetic gesture without speech as well (Quinto-Pozos and Parrill, 2008).^6^

Recent research has shown great similarities between entity constructions used by signers and corresponding (observer viewpoint) gestures used by non-signers. Schembri et al. (2005) used the Verbs of Motion Production task (Supalla et al., n.d.) to elicit entity constructions from Auslan signers and gestures from non-signers without speech. They found a considerable difference between signers’ and non-signers’ use of handshapes, but relatively less difference between signers’ and non-signers’ use of movement and spatial arrangement of the two hands. Specifically, signers used a smaller, more restricted set of handshapes to represent various entities than the non-signers did. Schembri et al. argue that this provides evidence for Liddell’s (2003b) analysis of some entity constructions as lexical verbs, which they suggest may involve a linguistically specified handshape which fuses with gestural elements of location and possibly movement.

Quinto-Pozos and Parrill (2012) also found similarities between the strategies used by signers and non-signers for explaining scenes or events from short cartoons presented via video. The ASL explanations were compared to co-speech gesture versions reported in Parrill (2010). In the signer–gesturer comparison, ASL signers’ use of entity constructions for depicting an object in its entirety and/or the path of an object through space were most common for the events that elicited observer viewpoint gestures exclusively in the non-signer data. In addition, signers used their bodies in mimetic ways the most (including for the portrayal of handling and the configuration of a character’s torso and/or head) for those events where the co-speech gesturers used character viewpoint gestures. Unlike the co-speech gesturers, however, the signers commonly used both entity constructions and mimetic strategies for depicting aspects of all cartoon events. The co-speech gesturers tended to rely on one strategy or the other when describing specific events. This work suggests a strong parallel between the ways signers and non-signers portray information about characters, although it reinforces the notion that signers can take advantage of the simultaneous nature of signed language productions (e.g., by using an entity construction for portraying a character’s path while also engaging mimetic displays of the upper body for showing the actions of a character).

Brentari et al. (2012a) examined the use of entity and handling constructions produced by signers of Italian Sign Language (LIS) and American Sign Language (ASL) and entity and handling gestures produced by non-signing Italian and English speakers, in pantomime – i.e. without speech. The participants, including both children (4- and 8-year-olds) and adults, were asked to describe what they had seen in vignettes that depicted either static objects or the manual manipulation of objects. The analysis of handshape was based on Brentari’s notion of selected finger complexity (Eccarius, 2008). The signers (LIS and ASL) patterned similarly and the gesturers (from Italy and the US) patterned similarly to each other, but the signers differed from the gesturers. With regard to handling, Brentari et al. indicated that the gesturers exhibited higher selected finger complexity than the signers (in both Italy and the US). Brentari et al. suggested that these results may be attributed to the task: handshapes in gesturers exhibited higher selected finger complexity because handling has a more accessible type of iconicity. In other words, the gesturers may have attempted to more directly imitate the handling that they had witnessed in the vignettes, whereas the handling handshapes for signers were informed by handshapes within the inventories of their languages. This preliminary work suggests that there could be some differences between ways that signers and gesturers describe the handling of objects, at least in terms of finger complexity, and it echoes the work of Schembri et al. (2005) in that the signers draw on a more conventionalised set of depicting hand configurations compared to gesturers.

If we consider co-speech gesture in addition to gesture without speech, there may be some similarity between signers’ handling constructions and non-signers’ handling gestures. In a study of categorical perception of handling handshapes, Sevcikova (2010, in preparation) investigated experimentally whether the size of handled objects is encoded (and decoded) by means of discrete handshapes in BSL or whether the handshapes convey more analogue descriptions of size of handled or manipulated objects. In the first study, categorical perception (CP) was examined to determine if handshapes continuously varying in aperture are perceived in a categorical manner by deaf BSL signers and hearing non-signers. Results revealed that handshapes used to describe handling and manipulation of flat, rectangular objects (e.g. books) and cylindrical objects (e.g. jars) were perceived categorically by both deaf BSL signers and hearing non-signers, pointing away from the existence of linguistic (phonemic) categories for handling handshapes in BSL but instead towards a more conventionalised, gestural system shared by both deaf signers and hearing non-signers (Sevcikova and Cormier, submitted for publication). Sevcikova’s second study examined whether the continuous variation in size of objects is categorically or discretely encoded in handling constructions produced by deaf BSL signers and hearing non-signers. Participants described handling of flat (rectangular) and cylindrical objects continuously increasing in size in thickness and diameter during narrative. Another group of deaf or hearing judges matched these handshapes back with the original item. Correlations between items described by the producers and items chosen by the judges were significant overall across both item continua for signers and non-signers. Closer inspection of data from deaf signers and hearing speakers revealed that within hypothesised categories of graspable object sizes (following Goldin-Meadow et al., 2007), the judges were at chance matching items with handshapes, which suggests somewhat categorical encoding of size for both object types. In contrast, hearing participants judging handshapes produced during pantomime displayed continuous size encoding for both object types. Thus, following Brentari et al. (2012a), Sevcikova found that signers displayed handling handshape categories while non-signers without speech (in pantomime condition) did not. However, unlike Brentari et al. who did not examine depicting handshapes in co-speech gesture, Sevcikova’s findings suggest that signers and non-signers using co-speech gesture both have conventionalised handshape categories for handling constructions. The fact that handling constructions used by signers are more similar to non-signers when speech and gesture are taken together as a package rather than when gesture is used without speech highlights the importance of the multimodal nature of face-to-face communication. Just as speech and co-speech gesture work in tandem, the same symbiotic relationship may be true of the combination of lexical signs and DCs: in both cases, the nominal lexical items(s) identifies the referent(s) and this may allow the use of referent-tracking depicting handshapes to be more schematic and categorical.

In handling gestures used by non-signers, the hand of the gesturer typically represents the hand of a referent; McNeill (1992) refers to these gestures as character-viewpoint gestures. But character-viewpoint gestures may additionally involve the use of non-manual articulators such as the head, face, torso, arms and hands to represent the same articulators of the referent. This is similar to a device known as constructed action in signed languages in which a signer uses various parts of his/her body to depict the actions, thoughts, and/or expressions of a character. Constructed action has been described as gestural in nature by some (e.g., Liddell and Metzger, 1998; Quinto-Pozos and Mehta, 2010), although the same productions have also been considered by others to be part of an exclusively linguistic system (e.g. ‘body classifiers’ described by Supalla, 1982, 1986, 2003). Most sign language researchers (including those who view constructed action as largely gestural in nature) consider constructed action to be different in some ways from character viewpoint gesture as used by non-signers. However, such differences may be more quantitative than qualitative in nature. For instance, signers typically make more use of constructed action during narratives than non-signers (Casey and Emmorey, 2009; Earis, 2008; Earis and Cormier, submitted for publication; Rayman, 1999). In any case, there can be strong similarities between productions of constructed action by signers and corresponding (character viewpoint) gestures produced by non-signers (Earis, 2008; Earis and Cormier, submitted for publication; Quinto-Pozos and Parrill, 2008, 2012; Brentari et al., 2012b). This is true for productions of the face, head and body, but also equally true for productions of the arms and hands, including handling constructions.

These synchronic similarities between DCs and some types of gesture suggest that DCs have gestural origins. This is quite different from verbal classifiers in spoken languages which are typically derived via grammaticalisation from lexical nouns (Grinevald, 2000; Mithun, 1986). Depicting handshapes in signed languages do not necessarily share any formational properties with their nominal referents. For example, the DCs in Figs. 1 and 5a above share no phonological properties with the BSL noun signs MAN and CAR, respectively. Instead, it is likely that depicting handshapes – both in DCs and in lexical signs –have developed (possibly simultaneously) as iconic representations of entities (especially in terms of shape) and handling/manipulation, as described in Section 3.^7^

To summarise, entity constructions in signed languages such as BSL, ASL and Auslan are extremely similar to observer viewpoint gestures used by non-signers, at least in location and movement. The greatest differences between entity constructions and observer viewpoint gestures are in the handshape inventories. Signers largely use a restricted, conventionalised, linguistically-defined set of handshapes with entity constructions but non-signers use much more idiosyncratic handshapes in their observer viewpoint gestures. Handling constructions look very similar in many respects to manual character viewpoint gestures used by non-signers.

## 5 Entity and handling constructions within the sign language lexicon

Thus far, we have shown parallels between entity constructions and observer viewpoint gestures and also between handling constructions and character viewpoint gestures. These parallels suggest that entity and handling constructions have gestural origins but of different types. Determining the degree of lexicalisation of particular constructions when working with natural sign language data can be very difficult (Janzen, 2012; Johnston and Schembri, 1999); therefore, we suggest that considering these different gestural origins can be useful in making such decisions. First, we consider handling constructions.

**Fig. 11:**
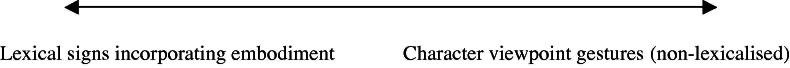
Continuum of lexicalisation of embodiment (including handling and constructed action).

In the analysis of discourse data, determining the difference between (a) an instance of a mimetic character viewpoint gesture/constructed action produced by the hands versus (b) the articulation of a handling construction versus (c) the use of a lexicalised sign using a handling handshape can be problematic. By considering character viewpoint gesture as a possible source for handling and other lexical signs of embodiment, we propose that handling constructions and constructed action are best thought of as existing along a continuum, shown in Fig. 11, with non-lexicalised character viewpoint gesture on one end and lexical signs of embodiment including handling (such as IPSL/BSL NEWSPAPER) on the other. A particular token of a handling construction or constructed action could fall anywhere along this continuum. Note that the lexical end of Fig. 11 refers to lexical signs of embodiment and not just handling. This is to account for lexicalisation of other embodied actions, not just those of the hands. Examples include the BSL signs CUDDLE (see Fig. 15 below) and BALLROOM-DANCE which each include a twisting movement of the torso (Brien, 1992).

**Fig. 12:**
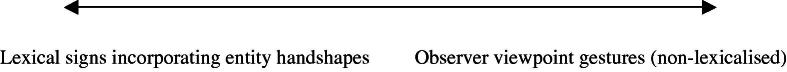
Continuum of lexicalisation of entity constructions (including signs and gestures depicting entities).

Distinguishing between entity constructions as used by signers and corresponding (observer viewpoint) gestures as produced by non-signers is more straightforward than identifying reliable differences between handling constructions produced by the two groups, as the set of entity handshapes within signed languages is more limited and may be quite different from the handshapes produced by hearing non-signers, as described above in Section 3. In addition, entity constructions and observer viewpoint gestures portray information in a small-scale, whereas handling constructions depict a life-sized scale of reference. Thus for entity constructions, we propose a separate continuum shown in Fig. 12, with observer viewpoint gestures on one end and lexicalised entity constructions (e.g. ASL FALL) on the other. As with the embodiment continuum in Fig. 11, a particular instance of an entity construction could fall anywhere along the continuum.

For both Figs. 11 and 12, on the gestural end of the continuum, productions are less constrained in terms of size and use of space, and less conventionalised in meaning. On the lexical side of the continuum, constructions are more constrained in terms of size and use of space, and more conventionalised in meaning. Both of these continua (and their characteristics) are reminiscent of the so-called ‘Kendon’s continuum’ of gesture and language which shares similar features (McNeill, 1992). However, one of the main characteristics of ‘Kendon’s continuum’ is differing degrees of reliance on speech (e.g. gesticulation relies heavily on speech while use of emblems does not). Clearly this characteristic is not relevant for sign languages. Instead, the two continua proposed here account for the two main types of DCs and their different gestural origins.

**Fig. 13:**
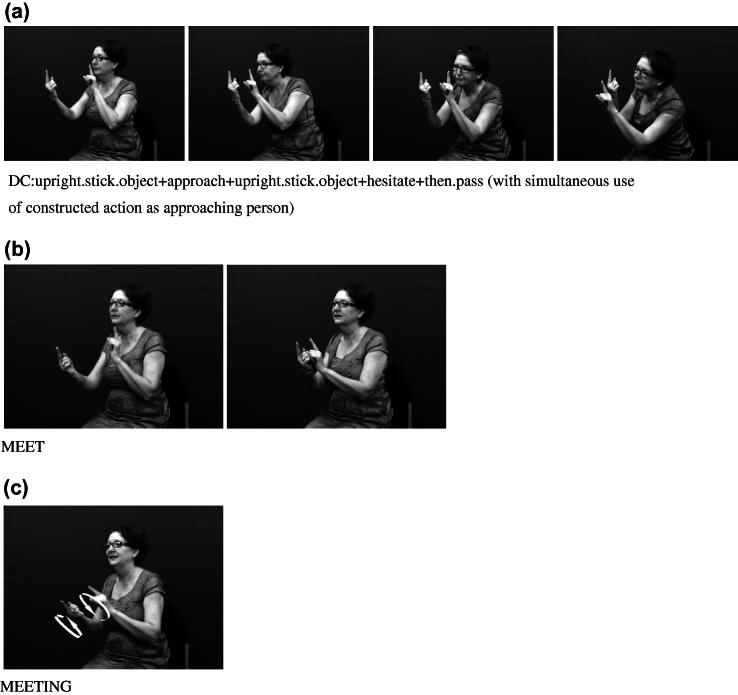
BSL entity handshapes in varying degrees of lexicalisation.

**Fig. 14:**
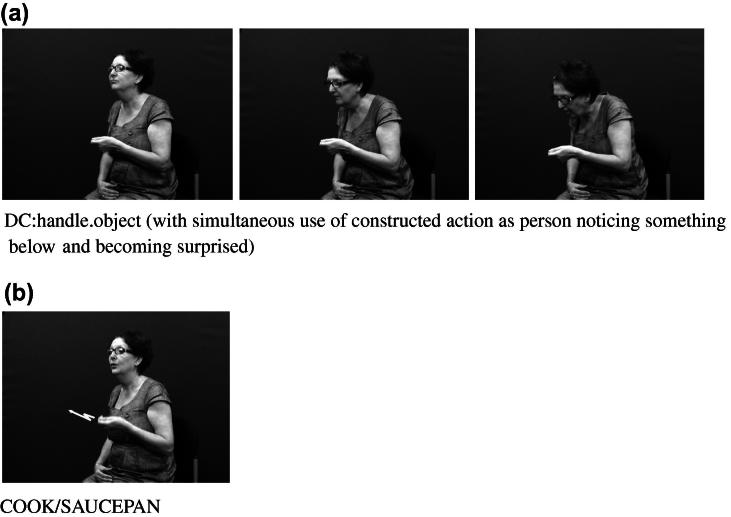
BSL handling handshapes in varying degrees of lexicalisation.

**Fig. 15:**
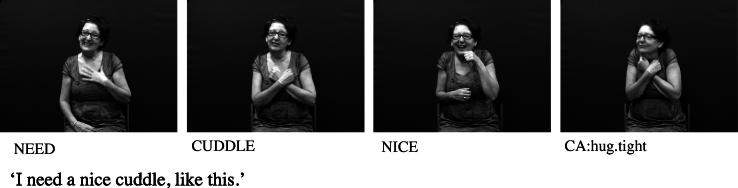
Sentence using both BSL embodied sign CUDDLE and related constructed action (CA).

**Fig. 16:**
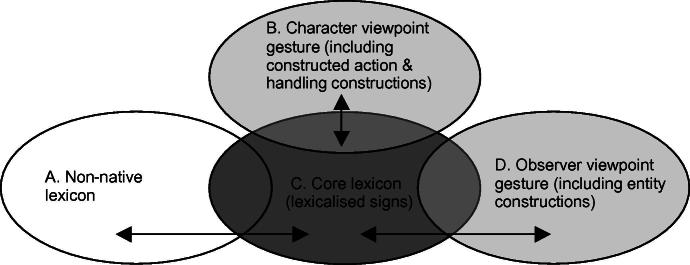
Model of the sign language lexicon (revised).

Considering both of these continua could help tease apart the various factors that can make it difficult to determine whether particular tokens of productions with depicting (entity or handling) handshapes are primarily lexical signs or depicting constructions or gestural representations. For example, in BSL and in many other signed languages, the entity handshape /index/ can be used in an entity construction to represent a stick-shaped figure (commonly in an upright position as an upright animate being), as in Fig. 13a which uses two /index/ entity handshapes, one approaching the other, hesitating and then moving past. Furthermore, signs which incorporate these two entity handshapes /index/ include the BSL lexical verb MEET, as in Fig. 13b or the BSL noun MEETING with alternating circular movements as in Fig. 13c. The BSL sign which incorporates the handling handshape /intl-T/, as if holding the long handle of a saucepan used for cooking can be extended to other referents as in Fig. 14a (in which the handled object could be anything that is typically handled that way, such as a conductor’s baton, a magnifying glass or a fan) or as a lexical item (a plain verb, COOK, or a noun SAUCEPAN), as in Fig. 14b. Certain contextual cues could be considered when making judgements about the lexical status of tokens of potential depicting constructions in signed languages. These include use of the token in a syntactic slot appropriate for a particular lexical category (e.g. the sign WANT followed by use of the sign in Fig. 13b to mean ‘want to meet’, or the sign WITH or WANT followed by use of the sign in Fig. 14b to mean ‘with a saucepan’ or ‘want to cook’), mouthing with the token (e.g. mouthing of the English words ‘meet’ or ‘saucepan’ or ‘cook’ simultaneously with the corresponding construction), and/or semantic extension (e.g. MEET used to refer to three or more people meeting, or COOK used to refer to cooking that involves cookware other than a saucepan).^8^ Cues for gestural status differ for entity and handling constructions – this is another motivation for favouring a split non-core lexicon as in Fig. 16 below. Cues for gestural status of entity constructions could be the lack of specificity of handshape, such that the less conventionalised the handshape, the stronger the observer viewpoint gestural status. Cues for gestural status of handling/embodiment could be the overtness of constructed action used (as marked by the number of articulators used and/or degree to which the various articulators are active, as in Figs. 13a and 14a which have overt constructed action marked by the signer’s head, face and body), or the degree of iconicity between production and referent – such that the more overt the constructed action and/or the higher the iconicity between production and referent, the stronger the character viewpoint gestural status.^9^ In many cases, for both types of construction, a given token could lie somewhere in the middle of the continuum. As discussed in Section 3, signers do alternate between depicting constructions and lexical items containing depicting handshapes. It is equally likely that signers alternate between constructed action (via the hands and/or non-manual articulators) and lexical items which include these iconic, embodied representations – such as the BSL sign CUDDLE followed by more elaborate constructed action showing specific manner of cuddling, as in Fig. 15. With both constructions of embodiment (i.e. within character perspective) and constructions in which the hand represents a referent (i.e. within observer perspective), signers may choose forms which may lie closer to the lexical end or to the gestural end of the continuum.

Considering Figs. 11 and 12 together allows us to go back and reconsider the model of the sign language lexicon as proposed by Brentari and Padden (2001) in Fig. 7 above. We propose that, at least in relation to DCs, Brentari and Padden’s ‘non-core lexicon’ is best considered as two separate parts of the lexicon, specifically Parts B and D shown in grey in Fig. 16. Parts B and D have different gestural origins and because of this the cues for lexicalised versus de-lexicalised status are different, as we have outlined here.

This split of the non-core lexicon has important implications for analysis of natural sign language data. The first step in coding or annotating sign language data is often to gloss the data at the lexical level, either by contextual word-for-sign glosses, or by ID glossing (Johnston, 2010). In either case, there is nearly always a need to identify tokens within the signing stream which are lexical signs (in the sense of the core lexicon) versus those which are not. Even for well-studied sign languages for which dictionaries exist, there are always tokens with entity or handling handshapes that occur in natural data, which could be considered lexicalised or not. Here we have attempted to provide some criteria which may be used in determining degree of lexicalisation, for constructions within both Part B and Part D of the lexicon.

## 6 Interaction between viewpoints in signers and gesturers

Although we have argued that Part B and Part D have different gestural origins, it should be noted that the two viewpoints do interact, and occur simultaneously, with each other in both signers and non-signers. The simultaneous use of constructed action and entity constructions in signed languages is well documented (e.g., Aarons and Morgan, 2003; Dudis, 2004; Perniss, 2007; Quinto-Pozos, 2007; Sallandre, 2007; Slobin et al., 2003). For example, entity construction(s) may be produced by the hand(s) while elements of constructed action are produced by articulators such as the face, head, and/or body, as in Fig. 13a above.

Non-signers also produce gestures in which character and observer viewpoints are expressed simultaneously. McNeill (1992) refers to these as ‘dual viewpoint iconic gestures’. McNeill (1992) describes an example from an elicited narrative where a /fist/ handshape is used to indicate Sylvester grabbing Tweety Bird (a character viewpoint gesture, where the gesturer’s hand represents Sylvester’s hand), and then a series of dual viewpoint gestures follow, where that same /fist/ handshape (still character viewpoint) moves downward to represent both Sylvester and Tweety falling, from an observer viewpoint.^10^ Parrill (2009) extends McNeill’s use of the term ‘dual viewpoint gestures’ to also include not only manual gestures but also combinations of manual and non-manual gestures, more similar to the simultaneous use of constructed action and entity constructions in signed languages as noted above.

## 7 Conclusion

Here we have shown that entity and handling constructions used by signers share many properties with observer viewpoint gestures and character perspective gestures used by non-signers, respectively. We have argued that, because of these different gestural origins, different types of cues are needed to distinguish the lexicalised versus de-lexicalised status of entity and handling constructions within the lexicon. When working with sign language data, a particular token of an entity construction or handling construction may be fully lexicalised, not at all lexicalised, or somewhere in between. Our model of the sign language lexicon proposed here recognises the rich gestural influence on depicting constructions but also the continuous bi-directional interaction between gestural and linguistic (e.g. lexicalisation) processes.

## Figures and Tables

**Figure:**
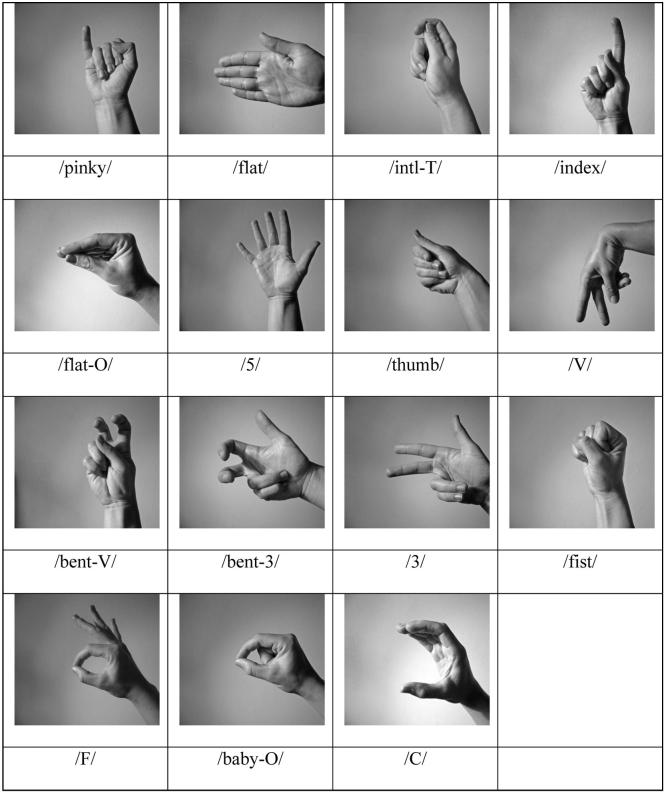


**Fig. 5a:**
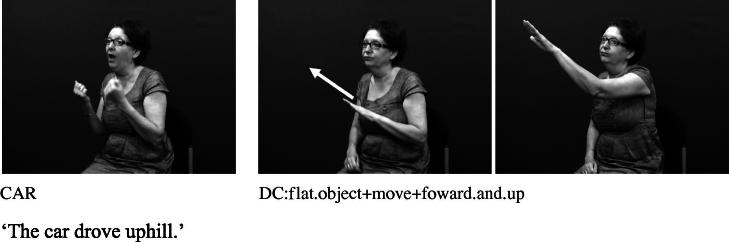
Entity handshape unit in BSL representing car.

**Fig. 5b:**
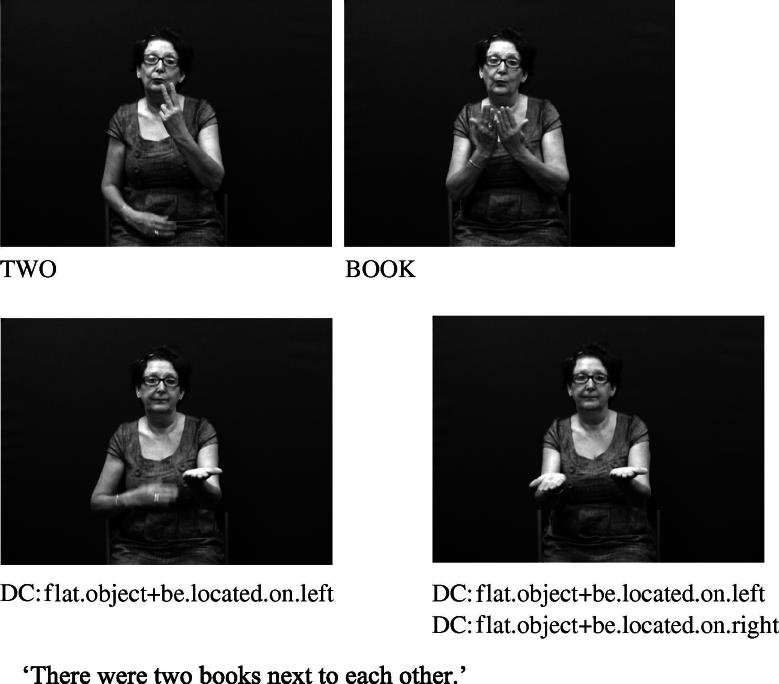
Entity handshape units in BSL representing books.

**Fig. 5c:**
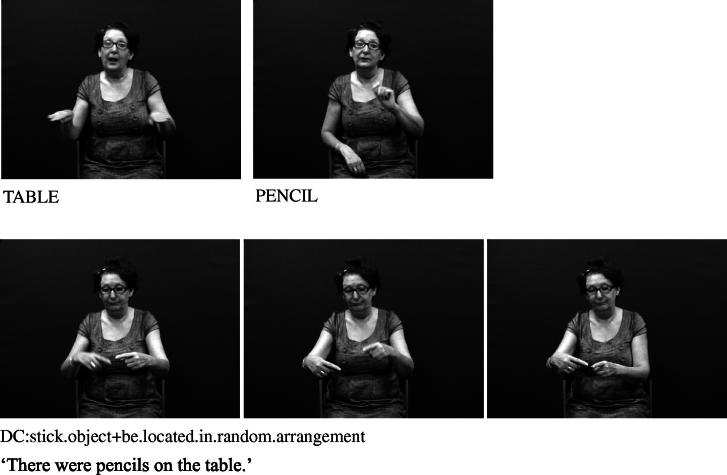
Entity handshape units in BSL representing pencils.

**Fig. 5d:**
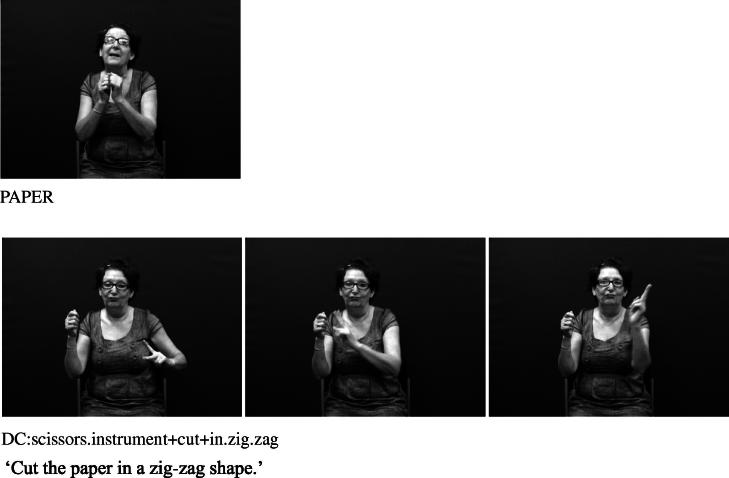
Entity handshape unit in BSL representing scissors.

**Fig. 6a:**
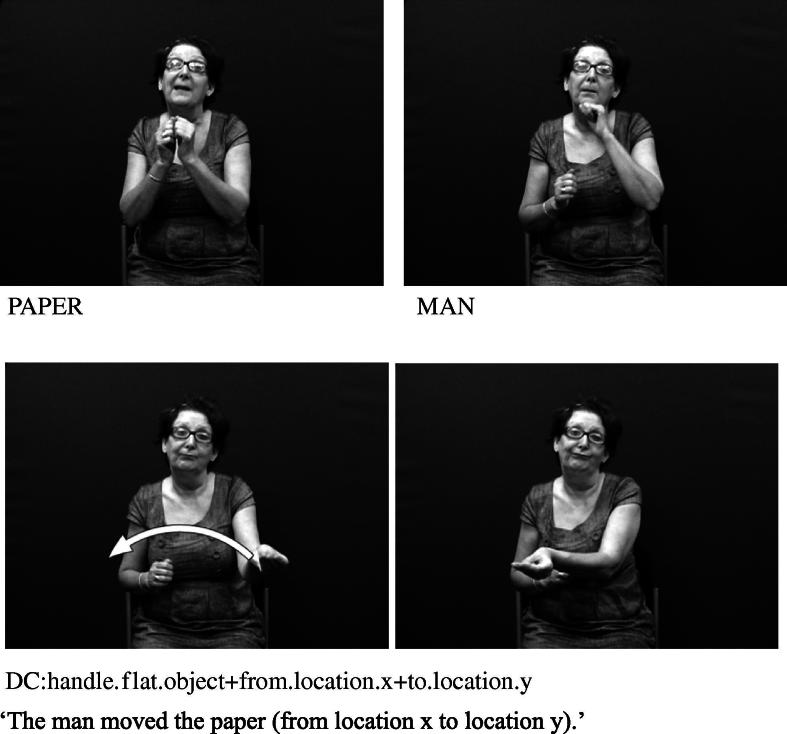
Handling handshape unit in BSL representing manipulation.

**Fig. 6b:**
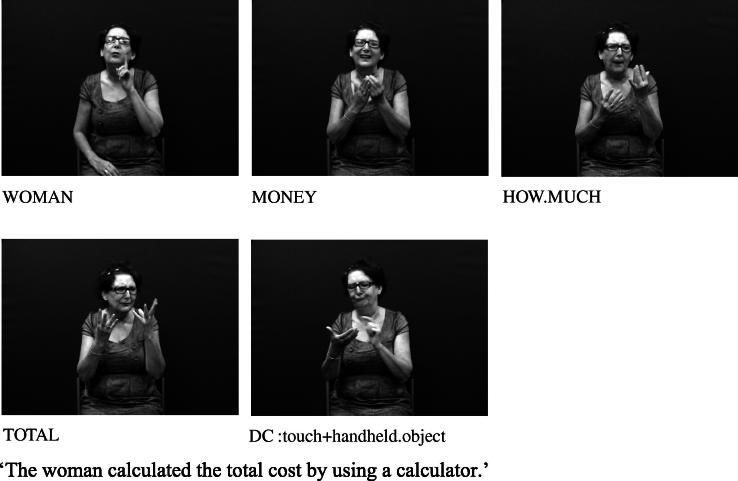
Handling handshape unit in BSL representing touching.

**Fig. 8a:**
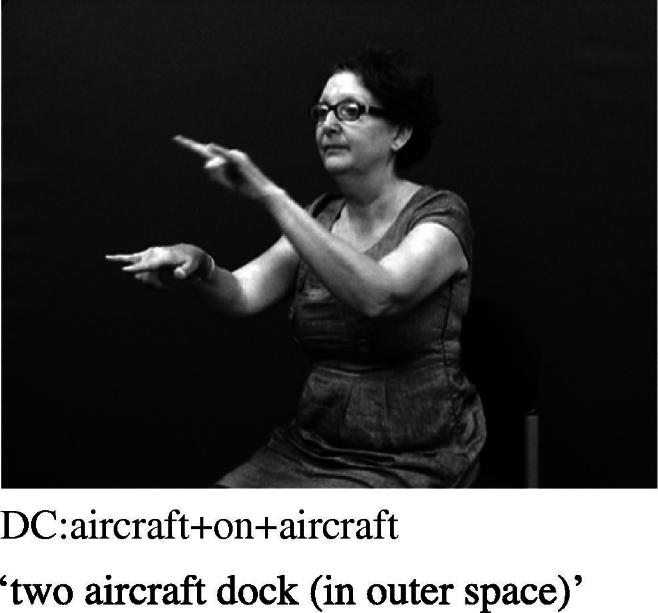
Entity handshapes representing two aircraft docking (ASL).

**Fig. 8b:**
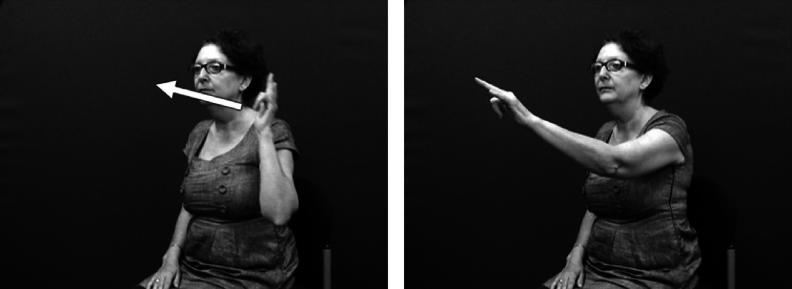
Entity handshape representing aircraft in lexical verb sign FLY.BY.PLANE (ASL).

**Fig. 8c:**
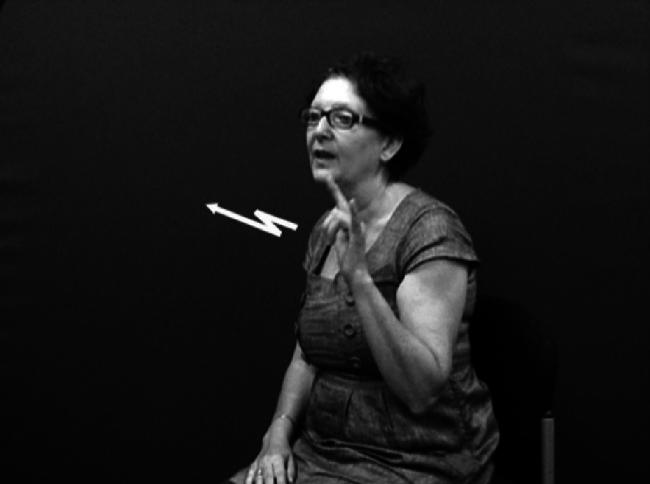
Entity handshape representing aircraft in lexical noun sign AIRPLANE (ASL).

**Fig. 9a:**
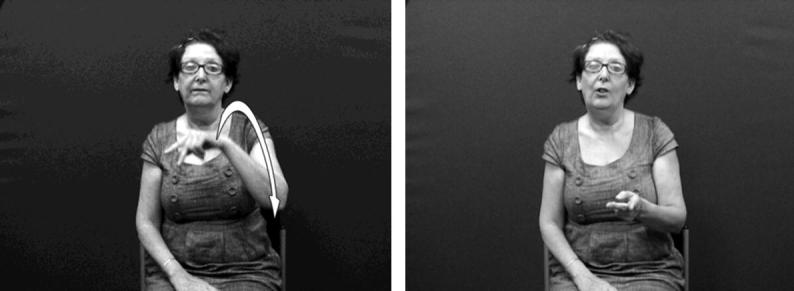
Lexical sign FALL (ASL/BSL).

**Fig. 9b:**
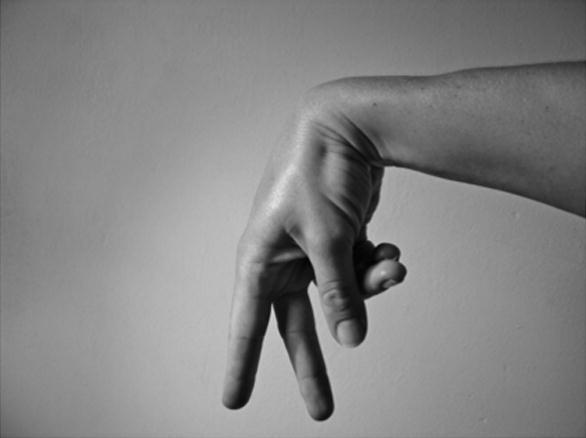
Biped /V/ entity handshape used in ASL/BSL FALL.

**Fig. 10a:**
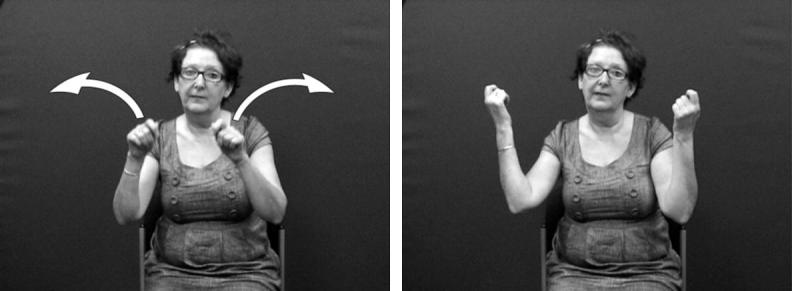
Lexical sign NEWSPAPER (IPSL/BSL).

**Fig. 10b:**
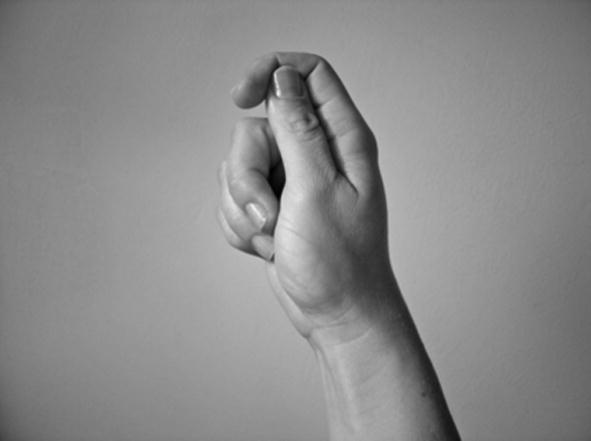
/intl-T/ handling handshape used in IPSL/BSL NEWSPAPER.

## Footnotes

1 Leipzig Glossing Rules (Bickel et al., 2008) are followed for spoken and signed language examples in this paper, adapted from the cited source where necessary. (Errors in glossing may be our own.) To our knowledge, this is the first time Leipzig Glossing Rules have been applied to sign language data, and some adaptations are needed. As is conventional in the sign language literature, lexical signs are glossed in capitals. Phonemic handshapes (illustrated in the Appendix) are labelled with a descriptive English word in lower case letters within phonemic slashes, e.g. /index/. Depicting constructions are glossed as ‘‘DC:’’ followed by a brief description in lower case letters. Constructed action is glossed as ‘‘CA:’’ followed by a brief description in lower case letters (see Section 4). A plus sign ‘+’ is used to distinguish meaningful elements within DCs and CA for which morphemic status is unclear.

2 In cases of DC systems with more than three categories, (e.g. 4, 5, 6 and 7-way distinctions), the types are mostly variations on or subcategorisations of the basic 2 or 3-way distinction. Interestingly, Kendon (2004, p. 160) notes a similar tripartite division in iconic co-speech gestures used by non-signers: modelling gestures in which a body part represents an object (cf. I), enactments or pantomime gestures in which the gesturing body parts enact corresponding body parts of a referent (cf. II) and depiction gestures in which body parts sculpt or trace the shape of an object in the air (cf. III).

3 Others do not view DCs from a linguistic perspective at all but instead propose that they are discrete units of a visual schematic representation with a consistent relationship between meaning and form, similar to conventions found in drawing and other forms of art (e.g. Cogill-Koez, 2000).

4 Fingerspelling is the use of a particular set of “signs” which each correspond to a different letter of the written alphabet of the surrounding spoken language. For more on fingerspelling and particularly nativisation of fingerspelling in ASL and in the British Sign Language family, see Cormier et al. (2008).

5 Brentari and Padden (2001) suggest that the non-core lexicon comprises not only entity and handling constructions but also size-and-shape specifiers, pointing (including pronominal and locative) signs and spatial verbs – specifically, vocabulary which “are understood in terms of their origin in the gestural domain” (p. 90). The relationship between the non-core lexicon and the gestural domain is also reflected in the model of the sign language lexicon proposed by Johnston and Schembri (1999), where lexicalisation processes occur between gesticulations, gesture and mime on one side of the continuum, and monomorphemic lexemes on the other.

6 Observer viewpoint gestures and character viewpoint gestures correspond to Kendon’s (2004) “modelling” gestures and “enactment (or pantomime)” gestures, respectively.

7 This is different from grammaticalisation pathways that have been described for sign languages, where there is some historical evidence e.g. for American Sign Language that gestures may become grammatical morphemes through two different paths: one via a lexical morpheme and another via prosody or intonation (Wilcox, 2007).

8 Note that non-signers can use visible gestures that are grammatically integrated into a spoken language utterance and, although not subject to lexicalisation, such tokens can take on ‘language-like’ properties (McNeill, 1992).

9 There are many types of iconicity – i.e. form/meaning correspondence – in sign languages. When comparing iconicity of constructed action and character viewpoint gestures, we mean the degree to which a production is a faithful representation of the referent.

10 Perniss and Ozyurek (2008) describe similar simultaneous uses of entity and handling constructions in Turkish Sign Language. However, they suggest that this kind of simultaneous construction is crosslinguistically very rare; it did not occur in any of their German Sign Language data. We know of no other signed language that has documented this type of fused construction in adult signers, though this construction has been identified in BSL narratives produced by deaf non-native signing children at age 9 (Smith and Cormier, submitted for publication). It may be that in order to convey observer and character perspectives simultaneously, signed languages generally prefer manual entity constructions and non-manual constructed action instead.

## References

[b0005] Aarons D., Morgan R. (2003). Classifier predicates and the creation of multiple perspectives in South African Sign Language. Sign Language Studies.

[b0010] Aikhenvald A.Y. (2003). Classifiers: A Typology of Noun Categorization Devices.

[b0015] Allan K. (1977). Classifiers. Language.

[b0020] Aronoff M., Meir I., Padden C., Sandler W., Emmorey K. (2003). Classifier constructions and morphology in two sign languages. Perspectives on Classifier Constructions on Sign Language.

[b0025] Battison R. (1978). Lexical Borrowing in American Sign Language.

[b0030] Bickel B., Comrie B., Haspelmath M. (2008). Leipzig Glossing Rules: Conventions for Interlinear Morpheme-by-Morpheme Glosses.

[b0035] Brennan, M., 1990. Word Formation in British Sign Language, Unpublished doctoral dissertation, University of Stockholm, Stockholm.

[b0040] Brennan M., Brien D. (1992). An introduction to the visual world of BSL. Dictionary of British Sign Language/English.

[b0045] Brennan M., Ahlgren I., Bergman B., Brennan M. (1994). Pragmatics and productivity. Perspectives on Sign Language Usage: Papers from the Fifth International Symposium on Sign Language Research.

[b0050] Brennan M., Brentari D. (2001). Making borrowings work in British Sign Language. Foreign Vocabulary in Sign Languages: Cross-Linguistic Investigation of Word Formation.

[b0420] Brentari, D., 2001. Foreign Vocabulary in Sign Languages. Lawrence Erlbaum, Mahwah, NJ.

[b0060] Brentari D., Padden C.A., Brentari D. (2001). Native and foreign vocabulary in American Sign Language: a lexicon with multiple origins. Foreign Vocabulary: A Cross-linguistic Investigation of Word Formation.

[b0055] Brentari D., Coppola M., Mazzoni L., Goldin-Meadow S. (2012). When does a system become phonological? Handshape production in gesturers, signers, and homesigners. Natural Language and Linguistic Theory.

[b0425] Brentari D., Nadolske M., Wolford G. (2012). Can experience with co-speech gesture influence the prosody of a sign language? Sign language prosodic cues in bimodal bilinguals. Bilingualism: Language and Cognition.

[b0065] Brien D. (1992). Dictionary of British Sign Language/English.

[b0070] Cabrera J.C.M., Ramat A.G., Hopper P.J. (1998). On the relationships between grammaticalization and lexicalization. The Limits of Grammaticalization.

[b0075] Casey S., Emmorey K. (2009). Co-speech gesture in bimodal bilinguals. Language and Cognitive Processes.

[b0080] Cogill-Koez D. (2000). Signed language classifier predicates: linguistic structures or schematic visual representation?. Sign Language and Linguistics.

[b0085] Cormier K., Schembri A., Tyrone M.E. (2008). One hand or two? Nativisation of fingerspelling in ASL and BANZSL. Sign Language and Linguistics.

[b0090] DeMatteo A., Friedman L. (1977). Visual imagery and visual analogues in American Sign Language. On the Other Hand: New Perspectives on American Sign Language.

[b0095] Dudis P.G. (2004). Body partitioning and real-space blends. Cognitive Linguistics.

[b0100] Earis, H., 2008. Point of View in Narrative Discourse: A Comparison of British Sign Language and Spoken English. Unpublished doctoral dissertation, University College London.

[b0105] Earis, H., Cormier, K., submitted for publication. Point of View in British Sign Language and Spoken English Narrative Discourse: The Example of ‘The Tortoise and the Hare’.

[b0110] Eccarius, P.N., 2008. A constraint-based Account of Handshape Contrast in Sign Languages. Unpublished doctoral dissertation, Purdue University, West Lafayette, IN.

[b0115] Edmondson, W.H., 1990. Segments in signed languages: do they exist and does it matter? In: Edmondson, W.H., Karlsson, F. (Eds.), SLR ‘87 Papers from the Fourth International Symposium on Sign Language Research. Signum, Hamburg.

[b0120] Emmorey K. (2002). Language, Cognition, and the Brain: Insights from Sign Language Research.

[b0125] Emmorey K. (2003). Perspectives on Classifier Constructions in Sign Languages.

[b0130] Engberg-Pedersen E. (1993). Space in Danish Sign Language.

[b0135] Frishberg N. (1975). Arbitrariness and iconicity: historical change in American Sign Language. Language.

[b0140] Goldin-Meadow S., Mylander C., Franklin A. (2007). How children make language out of gesture: morphological structure in gesture systems developed by American and Chinese deaf children. Cognitive Psychology.

[b0145] Grinevald C., Senft G. (2000). A morphosyntactic typology of classifiers. Systems of Nominal Classification.

[b0150] Grinevald C., Emmorey K. (2003). Classifier systems in the context of a typology of nominal classification. Perspectives on Classifier Constructions in Sign Languages.

[b0155] Hopper P.J., Traugott E.C. (2003). Grammaticalization.

[b0160] Janzen T., Pfau R., Steinbach M., Woll B. (2012). Lexicalization and grammaticalization. Sign Language: An International Handbook.

[b0165] Janzen T., Shaffer B., Meier R.P., Cormier K., Quinto-Pozos D. (2002). Gesture as the substrate in the process of ASL grammaticization. Modality and Structure in Signed and Spoken Languages.

[b0170] Johnson R.E., Liddell S.K., Testen D., Mishra V., Drogo J. (1984). Structural diversity in the American Sign Language lexicon. Papers from the Parasession on Lexical Semantics.

[b0175] Johnston T. (1991). Autonomy and integrity in sign languages. Signpost.

[b0180] Johnston T. (2010). From archive to corpus: transcription and annotation in the creation of signed language corpora. International Journal of Corpus Linguistics.

[b0185] Johnston T., Schembri A. (1999). On defining lexeme in a signed language. Sign Language and Linguistics.

[b0190] Johnston T., Schembri A. (2007). Australian Sign Language: An Introduction to Sign Language Linguistics.

[b0195] Kegl J., Wilbur R.B. (1976). Where does structure stop and style begin? Syntax, morphology, and phonology vs. stylistic variation in American Sign Language. Chicago Linguistic Society.

[b0200] Kendon A. (2004). Gesture: Visible Action as Utterance.

[b0205] Liddell S.K. (2003). Grammar, Gesture and Meaning in American Sign Language.

[b0210] Liddell S.K., Emmorey K. (2003). Sources of meaning in ASL classifier predicates. Perspectives on Classifier Constructions in Sign Languages.

[b0215] Liddell, S.K., Johnson, R.E., 1987. An analysis of spatial-locative predicates in American Sign Language. In: Paper Presented at Fourth International Conference on Sign Language Linguistics, Lapeenranta, Finland.

[b0220] Liddell S.K., Metzger M. (1998). Gesture in sign language discourse. Journal of Pragmatics.

[b0225] McDonald, B.H., 1982. Aspects of the American Sign Language Predicate System. Unpublished doctoral dissertation, State University of New York at Buffalo, Buffalo, NY.

[b0230] McDonald, B.H., 1985. Productive and frozen lexicon in ASL: an old problem revisited. In: Stokoe, W., Volterra, V. (Eds.), SLR ‘83: Proceedings of the 3rd International Symposium on Sign Language Research. CNR, Rome, pp. 254–259.

[b0235] McNeill D. (1992). Hand and Mind: What Gestures Reveal about Thought.

[b0240] Mithun M., Craig C. (1986). The convergence of noun classification systems. Noun Classes and Categorization.

[b0245] Padden C.A. (1998). The ASL lexicon. Sign Language and Linguistics.

[b0250] Parrill F. (2009). Dual viewpoint gestures. Gesture.

[b0255] Parrill F. (2010). Viewpoint in speech–gesture integration: Linguistic structure, discourse structure, and event structure. Language and Cognitive Processes.

[b0260] Perniss, P., 2007. Space and Iconicity in German Sign Language (DGS). MPI Series in Psycholinguistics vol.45. Radboud University, Nijmegen, Netherlands.

[b0265] Perniss P., Ozyurek A., Quer J. (2008). Constructing action and locating referents: a comparison of German and Turkish Sign Language narratives. Signs of the Time. Selected Papers from TISLR 8.

[b0270] Perniss P., Thompson R., Vigliocco G. (2010). Iconicity as a general property of language: evidence from spoken and signed languages. Frontiers in Psychology.

[b0275] Quinto-Pozos D. (2007). Why does constructed action seem obligatory? An analysis of “classifiers” and the lack of articulator-referent correspondence. Sign Language Studies.

[b0280] Quinto-Pozos D., Mehta S. (2010). Register variation in mimetic gestural complements to signed language. Journal of Pragmatics.

[b0285] Quinto-Pozos, D., Parrill, F., 2008. Enactment as a communicative strategy: a comparison between ASL and English co-speech gesture. In: Paper presented at 30th Annual Meeting of the German Linguistics Society, Bamberg, Germany.

[b0290] Quinto-Pozos, D., Parrill, F., 2012. Comparing viewpoint strategies used by co-speech gesturers and signers. In: Paper presented at 5th Conference of the International Society for Gesture Studies, Lund, Sweden.

[b0295] Rayman J., Winston E.A. (1999). Storytelling in the visual mode: a comparison of ASL and English. Storytelling and Conversation: Discourse in Deaf Communities.

[b0300] Sallandre M.-A., Vermeerbergen M., Leeson L., Crasborn O. (2007). Simultaneity in French Sign Language discourse. Simultaneity in Signed Languages: Form and Function.

[b0305] Sandler W., Lillo-Martin D. (2006). Sign Language and Linguistic Universals.

[b0310] Schembri, A., 2001. Issues in the analysis of polycomponential verbs in Australian Sign Language (Auslan). Unpublished doctoral dissertation, University of Sydney, Sydney.

[b0315] Schembri A., Emmorey K. (2003). Rethinking “classifiers” in signed languages. Perspectives on Classifier Constructions in Sign Languages.

[b0320] Schembri A., Jones C., Burnham D. (2005). Comparing action gestures and classifier verbs of motion: Evidence from Australian Sign Language, Taiwan Sign Language, and nonsigners’ gestures without speech. Journal of Deaf Studies and Deaf Education.

[b0325] Schick, B.S., 1987. The Acquisition of Classifier Predicates in American Sign Language. Unpublished doctoral dissertation, Purdue University, West Lafayette, IN.

[b0330] Seiler W. (1985). Imonda, a Papuan Language.

[b0430] Sevcikova, Z., 2010. Categorical perception and production of handling handshapes in BSL: evidence from BSL signing and hearing gesture. “Gesture – Evolution, Brain and Linguistic Structures” Paper presented at 4th Conference of the International Society for Gesture Studies, European University Viadrina Frankfurt/Oder, Germany.

[b0335] Sevcikova, Z., in preparation. Categorical versus Gradient Properties of Handling Handshapes in British Sign Language (BSL), Unpublished doctoral dissertation, University College London.

[b0340] Sevcikova, Z., Cormier, K., submitted for publication. Categorical perception of handling handshapes in British Sign Language: evidence from deaf signers and hearing non-signers.

[b0345] Shepard-Kegl, 1985. Locative relations in American Sign Language Word Formation Syntax and Discourse. Unpublished doctoral dissertation, Massachusetts Institute of Technology.

[b0350] Slobin D., Hoiting N., Kuntze M., Lindert R., Weinberg A., Pyers J., Anthony M., Biederman Y., Thurmann H., Emmorey K. (2003). A cognitive/functional perspective on the acquisition of “classifiers”. Perspectives on Classifier Constructions in Sign Languages.

[b0355] Smith, S., Cormier, K., submitted for publication. In or out? Use of spatial scale and enactment in narratives of native and non-native signing deaf children acquiring British Sign Language.

[b0360] Stokoe W. (1960). Sign Language Structure: An Outline of the Communication Systems of the American Deaf, Studies in Linguistics: Occasional Papers.

[b0365] Supalla T., Caccamise F., Hicks D. (1978). Morphology of verbs of motion and location. Proceedings of the Second National Symposium on Sign Language Research and Teaching.

[b0370] Supalla, T., 1982. Structure and Acquisition of Verbs of Motion and Location in American Sign Language. Unpublished doctoral dissertation, University of California at San Diego, San Diego, CA.

[b0375] Supalla, T., 1986. The classifier system in American sign language. In: Craig, C. (Ed.), Noun Classes and Categorization. John Benjamins, Philadelphia, PA, pp. 180-214.

[b0380] Supalla, T., 2003. Revisiting visual analogy in ASL classifier predicates. In: Emmorey, K. (Ed.), Perspectives on Classifier Constructions in Sign Languages, Mahwah, NJ, pp. 249–257.

[b0385] Supalla, T., Newport, E., Singleton, J., Supalla, S., Metlay, D., Coulter, G., n.d. The Test Battery for American Sign Language Morphology and Syntax, Unpublished Manuscript and Videotape Materials, University of Rochester, New York.

[b0390] Taub S., Galvan D. (2001). Patterns of conceptual encoding in ASL motion descriptions. Sign Language Studies.

[b0395] Wilcox S., Pizzuto E., Pietrandrea P., Simone R. (2007). Routes from gesture to language. Verbal and Signed Languages: Comparing Structures, Constructs and Methodologies.

[b0400] Zeshan U., Emmorey K. (2003). ‘Classificatory’ constructions in Indo-Pakistani sign language: Grammaticalization and lexicalization processes. Perspectives on Classifier Constructions in Sign Languages.

[b0405] Zwitserlood, I., 1996. Who’ll handle the object? An Investigation of the NGT Classifier, Unpublished Masters Dissertation, Utrecht University, Utrecht.

[b0410] Zwitserlood I. (2003). Classifying Hand Configurations in Nederlandse Gebarentaal (Sign Language of the Netherlands).

[b0415] Zwitserlood I., Pfau R., Steinbach M., Woll B. (2012). Classifiers. Sign Language: An International Handbook.

